# Analysis of Long Noncoding RNAs in Aila-Induced Non-Small Cell Lung Cancer Inhibition

**DOI:** 10.3389/fonc.2021.652567

**Published:** 2021-06-21

**Authors:** Lin Chen, Cui Wu, Heming Wang, Sinuo Chen, Danhui Ma, Ye Tao, Xingye Wang, Yanhe Luan, Tiedong Wang, Yan Shi, Guangqi Song, Yicheng Zhao, Xijun Dong, Bingmei Wang

**Affiliations:** ^1^ College of Clinical Medicine, College of Integrated Traditional Chinese and Western Medicine, Changchun University of Chinese Medicine, Changchun, China; ^2^ College of Animal Science, Jilin University, Changchun, China; ^3^ Department of Gastroenterology and Hepatology, Zhongshan Hospital, Fudan University, Shanghai, China; ^4^ Department of Gastroenterology and Hepatology, Zhongshan Hospital of Fudan University, Shanghai, China; ^5^ Affiliated Hospital to Changchun University of Chinese Medicine, Changchun University of Chinese Medicine, Changchun, China; ^6^ School of Pharmacy, Jilin University, Changchun, China

**Keywords:** Ailanthone, non-small cell lung cancer, long noncoding RNA, *DUXAP8*, *EGR1*

## Abstract

Non-small cell lung cancer (NSCLC) has the highest morbidity and mortality among all carcinomas. However, it is difficult to diagnose in the early stage, and current therapeutic efficacy is not ideal. Although numerous studies have revealed that Ailanthone (Aila), a natural product, can inhibit multiple cancers by reducing cell proliferation and invasion and inducing apoptosis, the mechanism by which Aila represses NSCLC progression in a time-dependent manner remains unclear. In this study, we observed that most long noncoding RNAs (lncRNAs) were either notably up- or downregulated in NSCLC cells after treatment with Aila. Moreover, alterations in lncRNA expression induced by Aila were crucial for the initiation and metastasis of NSCLC. Furthermore, in our research, expression of *DUXAP8* was significantly downregulated in NSCLC cells after treatment with Aila and regulated expression levels of *EGR1*. In conclusion, our findings demonstrate that Aila is a potent natural suppressor of NSCLC by modulating expression of *DUXAP8* and *EGR1*.

## Introduction

Lung cancer is the most widespread malignant tumor and has the highest mortality among all cancers. Based on one global cancer study conducted by the International Agency for Research on Cancer (IARC), there were approximately 4 million newly diagnosed and dead lung cancer patients in 2018 worldwide (1). Moreover, the number of people who are initially diagnosed with lung cancer is approximately 770,000, and those who die from lung cancer is nearly 700,000 annually in China (2). All of these data indicate that lung cancer is a tremendous threat to public health. Currently, lung cancer is classified into a variety of histological subtypes, among which NSCLC accounts for approximately 80-85% of all cases (3, 4). At present, the primary treatments for NSCLC include surgery, radiotherapy and pharmacotherapy, the latter including chemotherapy, targeted therapy, immunotherapy, etc. (5). However, due to the insidious onset of NSCLC, the majority of patients have lost the optimal timing for radical surgery at the time of diagnosis (6). In addition, since chemoradiotherapy has nonnegligible deficiencies, such as side effects, drug resistance and narrow indications (7, 8). Drug resistance and metastasis may arise during the chemotherapy, thereby substantially compromising the therapeutic efficacy of cancer treatment (9). So its overall therapeutic efficacy for NSCLC is unsatisfactory, and the 5-year survival rate of patients is poor at less than 20% (10). Therefore, it is particularly important to identify novel treatment method to provide early diagnosis, improve treatment efficiency, and reduce the mortality rate of NSCLC.

Currently, natural products have become a focus of new anticancer drug development, accounting for approximately 3/4 of clinical applications of antitumor drugs (11). Chinese herbal medicine is considered a gift of nature and these compounds derived from herbs have the advantage with availability, efficacy, and relatively low toxicity (12). As the primary active compound isolated from the root bark of the traditional medicinal plant Ailanthus altissima, Aila (11β,20-Epoxy-1β,11,12α-trihydroxypicrasa-3,13 (21)-diene-2,16-dione) has been proven to have a robust anticancer effect and can inhibit various cancers, including those arising in the reproductive system, urinary system, digestive system, blood system, respiratory system and other systems, in recent years (13). In genitourinary cancer, Aila significantly inhibited MDA-MB-231 mammary cancer cell viability and invasion and led to apoptosis by upregulating expression of miR-148a, blocking the AMPK and Wnt/β-catenin signaling pathways (14). Additionally, Wang et al. found that Aila induces apoptosis and restrains proliferation in MCF-7 mammary cancer cells by upregulating proapoptotic caspase-3 and upregulating the antiapoptotic apoptosis regulator B-cell lymphoma-2 (15). In addition, He et al. observed that Aila inhibited the proliferation and migration of castration-resistant prostate cancer (CRPC) cells and prevented drug resistance of the androgen receptor (AR) antagonist MDV3100 by binding *p23* (16). Daga et al. found that Aila also significantly reduced expression of *Nrf2*, *YAP* and *c-Myc* in 253J and T24 bladder cancer cells. Since these proteins can increase the drug resistance of cisplatin (CDDP), Aila plays a role in limiting the proliferation and migration of bladder cancer cells, as well as reversing drug resistance (17). Moreover, Cucci found that Aila inhibits the growth of A2780/CP70 oophoroma cells and reverses resistance to CDDP (18). For alimentary system cancers, Aila causes Huh7 hepatocellular carcinoma cell cycle arrest and induces apoptosis by downregulating cyclins and CDKs and upregulating *p21* and *p27* (19). Furthermore, Aila induced G (2)/M cell cycle arrest and apoptosis in SGC-7901 human gastric carcinoma cells by decreasing *Bcl-2* and increasing *Bax* expression (20). In terms of hematologic cancers, Wei et al. discovered that Aila exerts a tumor suppressor effect on HL-60 human promyelocytic leukemia cells and dose-dependently increases *beclin-1* and *LC3-II* and decreases *LC3-I* and *p62* expression (21). By upregulating *miR-449a* to disturb the Notch and PI3K/AKT signaling pathways, Aila represses acute myeloid leukemia (AML) cell metastasis and invasion (22). In lung cancer, Aila restrains cell proliferation and promotes apoptosis and autophagy by upregulating expression of miR-195 alone and reducing phosphorylation of *PI3K*, *Akt*, *JAK* and *STAT3* (23). Aila also inhibits DNA duplication to curb NSCLC cell growth by downregulating *RPA1* (24). Moreover, Aila exhibits inhibitory effects on other types of cancers. Liu et al. observed that Aila causes B16 and A375 melanoma cell cycle arrest and induces apoptosis, exerting a tumor-suppressive effect (25). Furthermore, Aila inhibits vestibular schwannomas (VS) by controlling miR-21 to regulate the Ras/Raf/MEK/ERK and mTOR signaling pathways (26). Aila also hinders MG63 osteosarcoma cell proliferation, migration, and invasion and induces apoptosis by upregulating miR-126 and downregulating *VEGF-A* to block PI3K/AKT signaling pathways (27).

LncRNAs, a class of RNAs with more than 200 nucleotides that perform essential regulatory functions with respect to genetic expression (28), are involved in the occurrence and development of numerous diseases, particularly tumors (29). With the development of profound experimental and high-throughput sequencing technology, a variety of lncRNAs have been identified as aberrantly expressed in NSCLC (30). For example, *MALAT1* is more highly expressed than in normal tissues in NSCLC, and its aberrant upregulation enhances the migration and invasion of NSCLC cells (31), while this effect was suppressed after implementation of gene silencing (32). In addition, *MEG3* promotes NSCLC cell proliferation by aberrant downregulation, the levels of which are correlated with the course of lung cancer, tumor size, and prognostic status (33) and strengthen the sensitivity of lung cancer cells to chemotherapeutic agents (34). Double homeobox A pseudogene 8 (*DUXAP8*), derived from a pseudogene (35), is highly expressed in many cancers, such as hepatocellular carcinoma (36), colorectal cancer (37) and oral cancer (38). Recently, Yin et al. determined that overexpression of *DUXAP8* in NSCLC cells not only promotes cell proliferation and migration but was also related to the clinical grade and prognosis of NSCLC patients, and downregulation of *DUXAP8* remarkably inhibited cell growth and migration (39).

Human early growth response factor-1 (*EGR1*) is a nuclear transcription factor belonging to the EGR family and containing a highly conserved DNA binding domain that binds to a GC-rich consensus sequence (40). In recent years, *EGR1* was proven to directly or indirectly upregulate multiple tumor suppressors, such as *PTEN*, *TP53*, *fibronectin*, *BCL-2* and *TGFb1* (40, 41), and was expressed at low levels in a variety of cancers, such as colon cancer (42) and oophoroma (43).

In this study, we found that Aila inhibits A549 and H1299 cell viability and invasion and promotes cell cycle stagnation and apoptosis. Moreover, exploring its molecular mechanism, we determined that *DUXAP8* was significantly downregulated and *EGR1* expression was upregulated in Aila-treated NSCLC cells. Moreover, knockdown of *DUXAP8* enhanced Aila’s antitumor effect, whereas its overexpression had the opposite effect. Consequently, these results indicate that Aila affects cell proliferation, cell cycle progression and apoptosis by reducing expression of *DUXAP8* to increase expression of *EGR1* in A549 and H1299 cells. Our research may provide new insight into therapeutic approaches for NSCLC.

## Materials and Methods

### Cell Culture

Human NSCLC A549 and H1299 cell lines were obtained from Jilin University. A549 cells were cultured in high glucose DMEM (HyClone, Los Angeles, USA), and H1299 cells were incubated in RPMI-1640 (HyClone, Los Angeles, USA). All culture media were supplemented with 10% fetal bovine serum (tbd Science, Tianjin, China) and 100 units/mL penicillin and streptomycin (HyClone, Los Angeles, USA) and were then cultured in a humidified atmosphere of 5% CO_2_ at 37°C.

### MTT Assay

The MTT assay was applied to determine the effect of Aila on NSCLC cell proliferation. Aila was purchased from BioBioPha Co., Ltd. (Yunnan, China). Briefly, A549 and H1299 cells were collected and seeded in 96-well plates at a density of 1×10^4^ cells per well. Following treatment with 1 μM Aila, MTT was added and incubated for another 4 h. The medium was changed to dimethyl sulfoxide (DMSO). A microplate reader was used to detect the optical density (OD) of the cells at 490 nm every 24 h until 72 hours.

### Live/Dead Cell Staining

Live/dead cell staining was used to visualize the influence of Aila on the viability of NSCLC cells. The Live and Dead Cell Double Staining Kit was obtained from Abbkine (Abbkine, Beijing, China). A549 cells were cultured in 24-well plates at 8 × 10^4^ cells per well. After administration of 1 μM Aila for 24 h, cells were stained for 15-30 min at room temperature in the dark according to the instructions. Subsequently, after washing cells with phosphate-buffered saline (PBS), they were imaged under a fluorescence microscope (Leica, Wetzlar, Germany) with appropriate filters as soon as possible.

### Colony Formation Assay

To test the role of Aila in A549 and H1299 cell tumorigenicity, a colony formation assay was performed. First, 100 cells/well were seeded into 6-well plates. Then, 1 μM Aila was added to the trial group, while an equal volume of solvent was added to the control group. Cells were cultured for 7 to 10 days, and the medium was replaced every 3 to 4 days. Furthermore, after washing with PBS, cells were fixed in 4% paraformaldehyde and stained with crystal violet. Finally, colonies were imaged and counted under an inverted microscope (Leica, Wetzlar, Germany).

### Wound Healing Assay

The wound healing assay was applied to assess the migration ability of NSCLC cells. Briefly, 7 × 10^4^ cells/well were cultured in 24-well plates. When A549 and H1299 cells reached 90% confluence, a wound area was created using a 200 μL pipette tip. Afterward, at 0, 24 and 48 h, images of cellular migration were captured.

### Transwell Assay

To evaluate the invasive capacity of A549 and H1299 cells, we performed a transwell assay. A total of 1 × 10^5^ cells per well were seeded into the upper transwell chamber precoated with 40 μL Matrigel and cultured in serum-free medium. Subsequently, 500 μL medium containing 10% FBS was transferred into the lower chamber. Cells continued to be incubated for 24 h. Subsequently, the upper cells were wiped off, and the invaded cells were fixed with 4% paraformaldehyde and stained with crystal violet. Images of stained cells were collected using an inverted microscope.

### Cell Cycle Analysis

We conducted cell cycle analysis using a Cell Cycle and Apoptosis Analysis Kit (Beyotime, Shanghai, China). In brief, A549 and H1299 cells were cultured in 6-well plates separately. Then, cells were treated with 1 μM Aila. Next, we fixed cells in 70% ethanol at 4°C for 12 to 24 h. Following washing and collection, cells were resuspended in 500 μL PI staining solution for 30 min in the dark. Ultimately, these dyed cells were detected using the PI signal detector of the flow cytometer (Beckman Coulter, USA), and the results were analyzed using ModFit LT.

### Cell Apoptosis Analysis

An Annexin V-FITC Apoptosis Detection Kit (Beyotime, Shanghai, China) was used to perform the apoptosis analysis. First, A549 and H1299 cells were seeded into 6-well plates and treated with 1 μM Aila. Then, cells were harvested and resuspended in 195 μL binding buffer along with 5 μL Annexin V-FITC and 5 μL PI. Flow cytometry was used to measure cell apoptosis, and FlowJo (vX.0.7) was employed to analyze the data. An One Step TUNEL Apoptosis Kit (Beyotime, Shanghai, China) was also employed according to the manufacturer’s instruction.

### LncRNA-seq and Data Analysis

We performed high-throughput lncRNA sequencing to further explore the molecular mechanism of Aila in NSCLC. Initially, H1299 and A549 cells were evenly cultured in 10 cm dishes. When cells reached 80-90% confluence, 3 μM Aila was added for 24 h, and the control group was set up. Subsequently, collected cells were sent to GENEWIZ Biotech (GENEWIZ, Suzhou, China) to perform lncRNA sequencing. Total RNAs were isolated using TRIzol solution. Then, next-generation sequencing library preparations were constructed using ribosomal depleted RNA. Therefore, sequencing was implemented on an Illumina NovaSeq (Illumina, San Diego, CA, USA) using a 2x150 paired-end (PE) configuration. Clean data were obtained after removing adapters and QCs less than 25 in raw sequencing date using trim_galore (0.6.4). Then, data were analyzed using STAR (STAR_2.6.1a) to map clean data to a reference human genome (GRCh38). After that, transcripts were quantified and annotated (GENECODE v35) using stringtie (1.3.3), and the count for each transcript was obtained. Furthermore, transcripts were normalized, and differential expression analysis was performed using DESeq2. Moreover, GO and KEGG enrichment analyses were performed using clusterProfiler (44). The data of LUAD patients were obtained from TCGA. Sequencing data were submitted to the Sequence Read Archive (SRA) dataset under the accession number PRJNA (PRJNA690710).

### Knockdown and Overexpression of *DUXAP8*

We knocked down and overexpressed *DUXAP8* to verify the regulatory role of lncRNAs in NSCLC. Si-*DUXAP8* was purchased from RiboBio (RiboBio, Guangzhou, China), and pcDNA3.1-*DUXAP8* was constructed in the laboratory. A549 and H1299 cells were collected when the density was approximately 70-90%. Subsequently, cells were transfected using Lipofectamine™ 3000 Reagent (Thermo Fisher, Massachusetts, USA) following the manufacturer’s instructions. Afterward, the medium was changed to serum-containing medium after 4 h of transfection. Moreover, RT-PCR was used to examine whether knockdown and overexpression were successfully established. The siRNA target sequences are listed in **Table S1**.

### Real-Time PCR

A549 cells were incubated in 6 cm dishes, and one group was treated with 1 μM Aila, while the other was given an equal volume of DMSO. First, total RNA was extracted from A549 cells using TriPure isolation reagent (Roche, Basel, Switzerland). Then, cDNA was synthesized using Plus All-in-one 1st Strand cDNA Synthesis SuperMix (Novoprotein, Shanghai, China) according to the manufacturer’s guidelines. Furthermore, quantitative real-time PCR was performed utilizing SYBR qPCR SuperMix Plus (Novoprotein, Shanghai, China) on a PIKOREAL 96 Real-Time PCR System. RT-PCR was conducted using the following parameters: predenaturation at 95°C for 1 min, followed by 40 cycles of denaturation at 95°C for 20 s, annealing at 60°C for 20 s, and extension at 72°C for 30 s. Moreover, GAPDH was used as an internal reference, and expression of related genes was calculated utilizing the 2^-ΔΔCt^ method. Primer sequences are listed in **Table S2**.

### Western Blot Analysis

First, A549 cells were collected and lysed in RIPA buffer to isolate proteins. Protein concentrations were subsequently determined using a BCA protein reagent assay kit (Beyotime, Shanghai, China). Next, 10% sodium dodecyl sulfate-polyacrylamide gel electrophoresis (SDS-PAGE) was conducted, followed by transfer of proteins onto a membrane. Afterward, membranes were blocked in 5% nonfat milk and then probed with primary antibodies against anti-EGR1 (Affinity, BF0443) and anti-GAPDH (Proteintech, 60004-1-Ig) at 4°C overnight. Furthermore, membranes were washed and incubated with horseradish peroxidase (HRP) and incubated with conjugated polyclonal goat antimouse IgG (Beyotime, A0216) secondary antibody for 1 h at room temperature. Finally, Fusion FX Edge Spectra (VILBER LOURMAT, Paris France) was utilized for imaging after washing the membranes once again.

### Mouse Xenograft Experiments

Female nude mice (4-5 weeks) were employed to perform the xenograft experiments. First, 10 mice were randomly divided into two groups, and each group was subcutaneously injected with 1 × 10^6^ H1299 cells. One week after tumor induction, mice in the trial group were intraperitoneally injected with 2 mg/kg Aila daily, while controls were treated with an equal volume of saline. Treatments were continued for 2 weeks. Subsequently, mice were sacrificed with CO_2_ asphyxiation.

### Statistical Analysis

In all experiments, trial and control groups were set up, and at least three independent experiments were performed. Data are expressed as the mean ± standard deviation (SD). Statistical analyses were performed using GraphPad statistical software (GraphPad Software, La Jolla, CA). P<0.05 was considered statistically significant.

## Results

### Aila Inhibits A549 and H1299 Cell Viability

**Figure 1:**
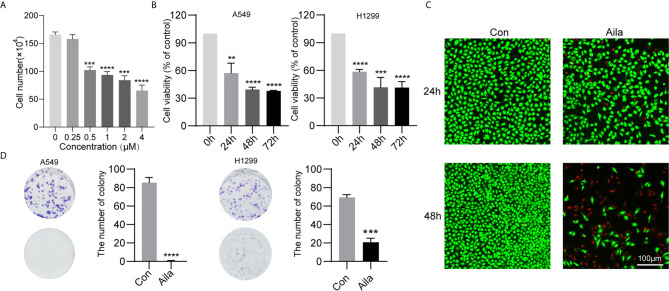
Aila inhibits A549 and H1299 cell viability and colony formation. **(A)** Cells treated with different dosages of Aila (0, 0.25, 0.5, 2, 2 and 4 μM). **(B)** The proliferation of A549 and H1299 cells was analyzed by MTT assay at 24, 48 and 72 h. **(C)** Cell viability of A549 cells was detected by live/dead assay after the addition of 1 μM Aila with an established control group. **(D)** Colony formation of A549 and H1299 cells was performed after treatment with or without 1 μM Aila in 6-well plates. Data are represented as the mean ± SD (n = 3). **(P < 0.01), ***(P < 0.001) and ****(P < 0.0001) indicate statistically significant differences.

To test the anticancer effect of Aila on NSCLC, we examined the cell growth and colony formation of A549 and H1299 cells. A549 cells were treatment with Aila and counted by blood counting chamber. We chose 1μM for subsequent experiments (**Figure 1A**). From the MTT assay, we could see that Aila significantly inhibited the proliferation of A549 and H1299 cells in a time-dependent manner (P<0.01) (**Figure 1B**). Additionally, Aila significantly restrained cell viability in live/dead cell staining (**Figure 1C**). Moreover, colony formation was reduced after treatment with Aila (**Figure 1D**).

### Aila Restrains A549 and H1299 Migration and Invasion

**Figure 2:**
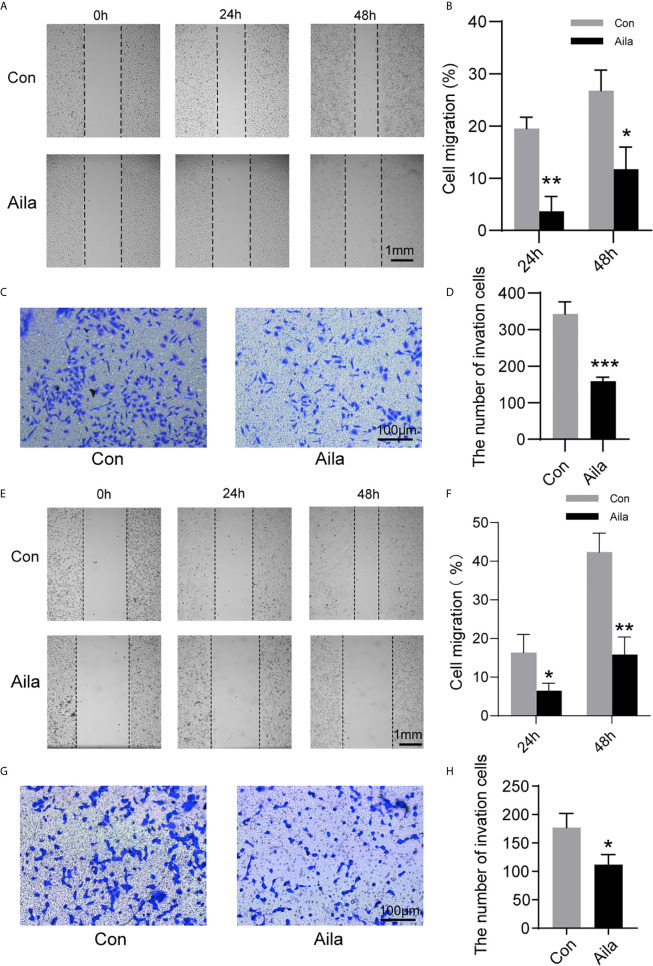
Aila restrains A549 and H1299 cells migration and invasion. **(A, B)** Cell migration of A549 cells was tested by wound healing experiments between control and Aila groups. **(C, D)** Cell invasion was analyzed using the transwell assay of A549 cells. **(E, F)** Cell migration of H1299 cells was tested by wound healing experiments between control and Aila groups. **(G, H)** Cell invasion was analyzed using the transwell assay of H1299 cells. The data are represented as the mean ± SD (n = 3). *(P < 0.05), **(P < 0.01) and ***(P < 0.001) indicate statistically significant differences.

Wound healing assays and transwell assays were performed to observe the influences of Aila on migration and invasion. Wound images were collected at 0, 24 and 48 h after the scratch was made. Results showed that Aila clearly slowed cell migration of A549 cells (P<0.05) (**Figures 2A, B**) and H1299 cells (P<0.01) (**Figures 2E, F**). Furthermore, compared to the control group, the inhibitory effect of Aila on cell invasion was significant of A549 cells (P<0.001) (**Figures 2C, D**) and H1299 cells (P<0.05) (**Figures 2G, H**).

### Aila Induces A549 and H1299 Cell Cycle Arrest and Apoptosis

**Figure 3:**
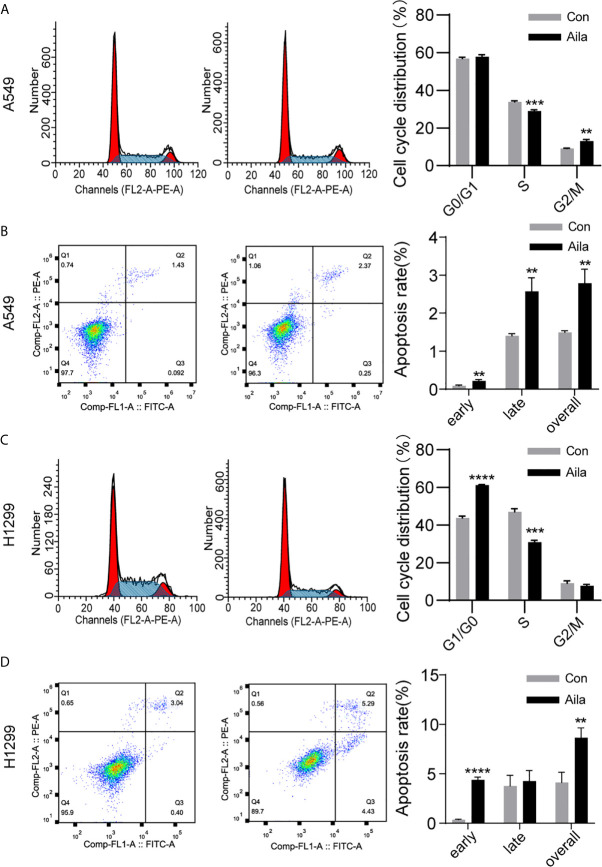
Aila induces cell cycle arrest and apoptosis in A549 and H1299 cells. **(A, C)** The cell cycle of A549 and H1299 cells was examined by flow cytometry between control and Aila groups. **(B, D)** Apoptosis of A549 and H1299 cells was analyzed between control and Aila groups and the quantified apoptosis data refers to the total proportion of both early and late apoptosis. The results were analyzed using FlowJo and ModFit software. Data are represented as the mean ± SD (n = 3). **(P < 0.01), ***(P < 0.001) and ****(P < 0.0001) indicate statistically significant differences.

The uncontrollable growth of tumors is primarily related to cell cycle disturbances (45). Therefore, cell cycle analysis was performed to test whether Aila had a positive effect on the cell cycle arrest. Flow cytometry results showed that the S phase was decreased in A549 and H1299 cells after treatment with Aila (P < 0.001) (**Figures 3A, C**). Therefore, we concluded that Aila led to G1 stagnation in A549 and H1299 cells.

To further investigate whether Aila suppressed the growth of A549 and H1299 cells by triggering apoptotic signals, we used Annexin V/PI double staining to evaluate the apoptotic effects of Aila on A549 and H1299 cells. Results revealed that apoptosis of A549 and H1299 cells was increased in response to treatment with Aila (P < 0.01) (**Figures 3B, D**), implying that Aila significantly induces apoptosis.

### Aila Downregulates *DUXAP8* in A549 Cells

**Figure 4:**
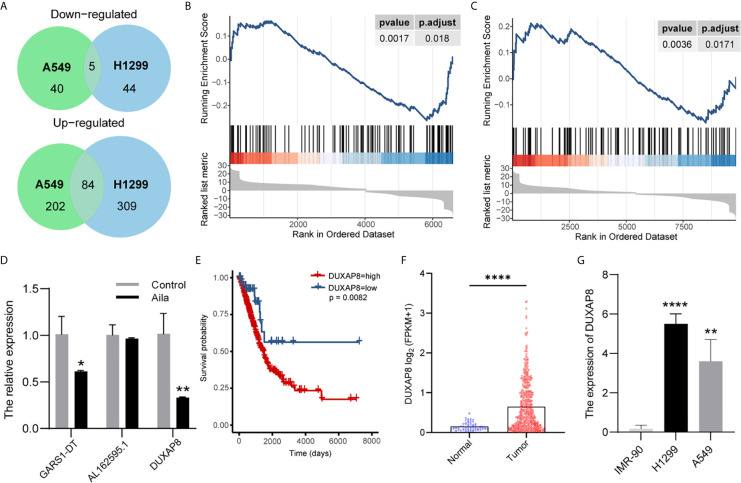
LncRNA expression profiling by RNA-seq. **(A)** Venn diagrams of differentially expressed lncRNAs. Gene set enrichment analysis (GSEA) revealed that apoptosis was upregulated after treatment with Aila in A549 cells **(B)** and H1299 cells **(C)**. **(D)** Relative expression of GARS1-DT, AL162595.1, and DUXAP8 was analyzed by RT-PCR after Aila treatment in A549 cells. **(E)** The effect of DUXAP8 expression level on patient survival in TCGA database. **(F)** Expression of DUXAP8 in LUAD based on sample types. **(G)** The expression of DUXAP8 between IMR-90, A549 and H1299 cells in CCLE database. Data are shown as the mean ± SD (n = 3). *(P < 0.05), **(P < 0.01) and ****(P < 0.0001) indicate statistically significant differences.

To explore lncRNA expression patterns after treatment with Aila in NSCLC, lncRNA-seq was performed with Illumina NovaSeq in H1299 cells. A total of 489 lncRNAs in A549 cells and 339 lncRNAs in A549 cells were differentially expressed between cells treated with Aila and untreated cells. From Venn diagrams, GARS1-DT, AL162595.1, DUXAP8, AC027627.1 and AC008735.2 were downregulated in two cell lines (**Figure 4A**). GSEA result show that genes involved in apoptosis were enriched after treatment with Alia in two cell lines (**Figures 4B, C**). Next, RT-PCR confirmed that DUXAP8 was significantly downregulated (**Figure 4D**). Moreover, The Cancer Genome Atlas (TCGA) database showed that *DUXAP8* in lung adenocarcinoma patients (LUAD) was significantly higher than in noncancerous tissue, and the level of *DUXAP8* upregulation was associated with poor prognosis and reduced survival (**Figures 4E, F**). The Cancer Cell Line Encyclopedia (CCLE) database revealed that *DUXAP8* in A549 and H1299 cells was significantly higher than in IMR-90 cells (**Figure 4G**). At present, one study has found that knockdown of *DUXAP8* inhibits growth of NSCLC cells (39). These data support our results, suggesting that Aila inhibits the growth of H1299 cells by downregulating *DUXAP8*.

### Effects of Knockdown and Overexpression of *DUXAP8* on Cell Viability and Invasion

**Figure 5:**
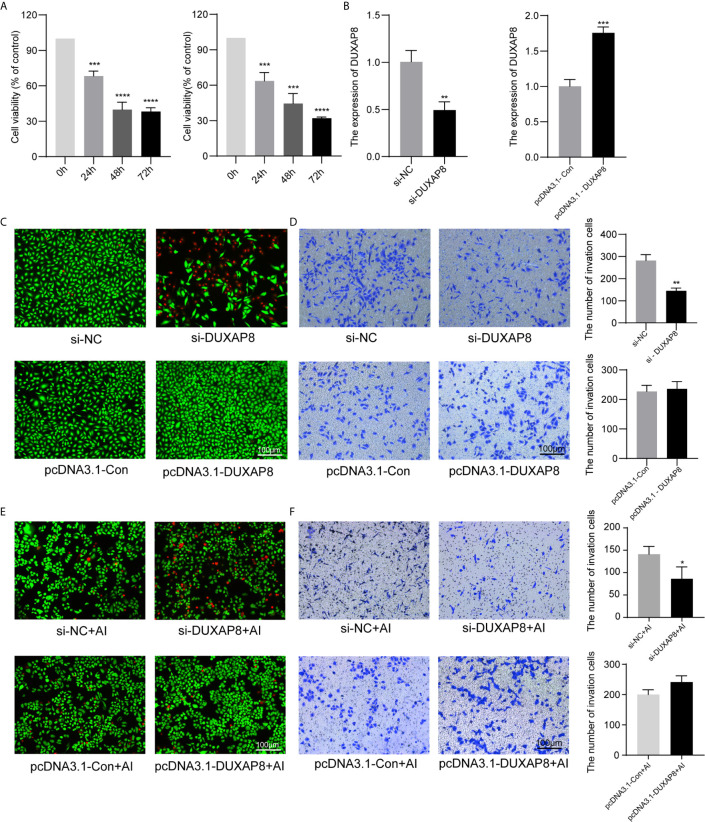
Analysis of the effects of *DUXAP8* expression on the viability and invasion ability of A549 and H1299 cells. **(A)** Cell growth of A549 and H1299 cells was analyzed after si-*DUXAP8* transfection by MTT assay. **(B)** Relative expression of *DUXAP8* in the Nc, si-*DUXAP8*, pcDNA3.1-Con and pcDNA3.1-*DUXAP8* groups using RT-PCR. **(C)** Live/dead cell staining of A549 cells was analyzed after si-*DUXAP8* and pcDNA3.1-*DUXAP8* transfection. **(D)** The invasion assay of A549 cells was performed after si-*DUXAP8* and pcDNA3.1-*DUXAP8* transfection. **(E)** Live/dead cell staining of A549 cells was analyzed after si-*DUXAP8* and pcDNA3.1-*DUXAP8* transfection and Aila treatment. **(F)** The invasion assay of H1299 cells was performed after si-*DUXAP8* and pcDNA3.1-*DUXAP8* transfection and Aila treatment. Data are shown as the mean ± SD (n = 3). *(P < 0.05), **(P < 0.01), ***(P < 0.001) and ****(P < 0.0001) indicate statistically significant differences.

A549 and H1299 cells were transfected with si-*DUXAP8* and pcDNA3.1-*DUXAP8* expression vectors and treated with Aila to further elucidate the possible regulatory mechanism of *DUXAP8* in NSCLC. RT-PCR revealed that the expression of *DUXAP8* was decreased in response to si-*DUXAP8*, while that in pcDNA3.1-*DUXAP8* was increased, indicating that knockdown and overexpression were successfully established (**Figure 5B**). Next, we investigated the phenotypes of NSCLC after knockdown and overexpression of *DUXAP8* and Aila treatment. Compared to the control group, si-*DUXAP8* significantly attenuated cell viability (**Figures 5A, C**). Aila and si-*DUXAP8* treatment also significantly attenuated cell viability (**Figure 5E**). Besides, si-*DUXAP8* significantly reduced the number of invaded cells, while overexpression of *DUXAP8* has no significant effects (**Figure 5D**). Cells treated with Aila and si-*DUXAP8* and overexpression of *DUXAP8* has the same trend (**Figure 5F**).

### Effects of Knockdown and Overexpression of *DUXAP8* on Cell Cycle and Apoptosis in A549 Cells

**Figure 6:**
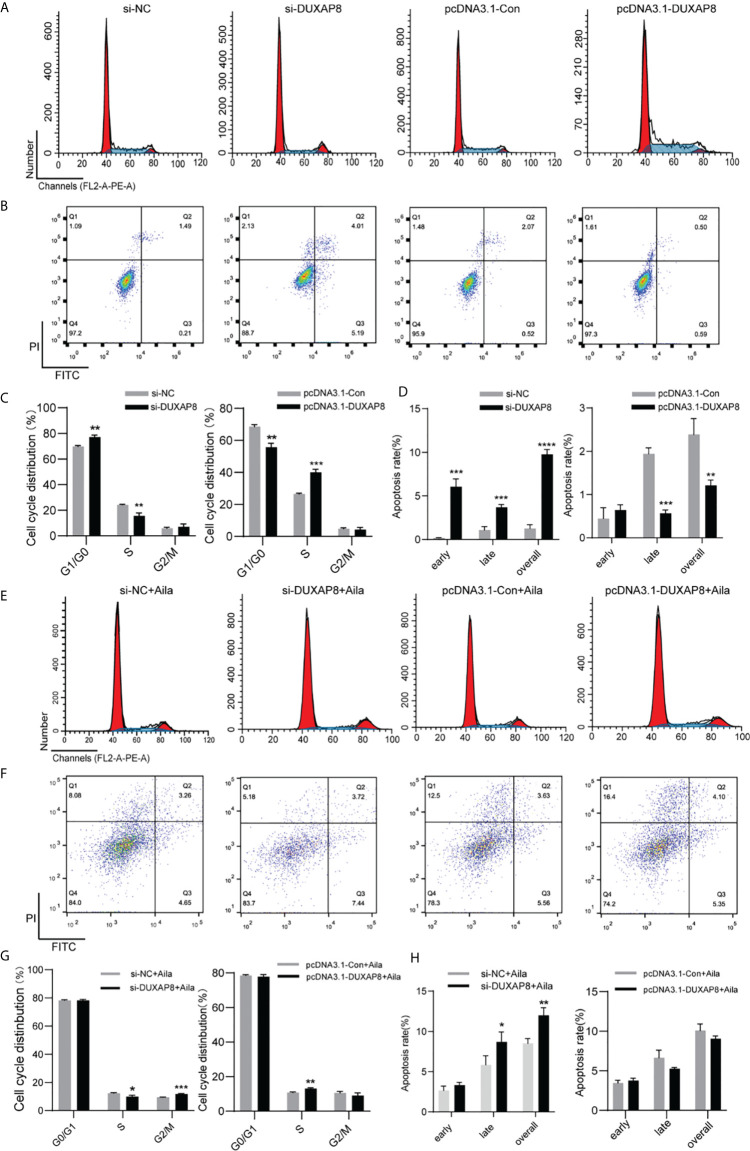
Analysis of the effects of *DUXAP8* expression on the cell cycle and apoptosis of A549 cells. **(A)** The cell cycle was analyzed after si-*DUXAP8* and pcDNA3.1-*DUXAP8* transfection of A549 cells. **(B)** Apoptosis was detected after si-*DUXAP8* and pcDNA3.1-*DUXAP8* transfection of A549 cells. **(C)** Statistical analysis of the percentage of cell cycle distribution. **(D)** The quantified apoptosis data were calculated from the total proportion including both early and late apoptosis, the histogram represents the sum of Q2 and Q3. **(E)** The cell cycle was analyzed after si-*DUXAP8* and pcDNA3.1-*DUXAP8* transfection and treated with Aila. **(F)** Apoptosis was detected after si-*DUXAP8* and pcDNA3.1-*DUXAP8* and treated with Aila. **(G)** Statistical analysis of the percentage of cell cycle distribution. **(H)** The quantified apoptosis data were calculated from the total proportion including both early and late apoptosis, the histogram represents the sum of Q2 and Q3. The data are represented as the mean ± SD (n = 3). *(P < 0.05), **(P < 0.01), ***(P < 0.001) and ****(P < 0.0001) indicate statistically significant differences.

To further analyze the effect of expression of *DUXAP8* on the cell cycle and apoptosis, A549 cells was transfected with si-*DUXAP8* or pcDNA3.1-*DUXAP8* expression vectors and treated with Aila. Flow cytometry results showed that si-*DUXAP8* significantly decreased S phase, while pcDNA3.1-*DUXAP8* reversed this pattern in A549 cells (**Figures 6A, C**). Cells treated with Aila and si-*DUXAP8* also significantly decreased S phase and overexpression of *DUXAP8* and Aila treated reversed this pattern (**Figures 6E, G**). To investigate whether *DUXAP8* is associated with apoptosis in A549 cells, we used the Annexin V/PI double staining method. Results revealed that si-*DUXAP8* induced apoptosis of A549 cells, while pcDNA3.1-*DUXAP8* reversed this effect (**Figures 6B, D**). Cells treated with Aila and si-*DUXAP8* also induced apoptosis and overexpression of *DUXAP8* and Aila treated reversed this pattern (**Figures 6F, H**).

### Knockdown and Overexpression of *DUXAP8* on Cell Cycle and Apoptosis in H1299 Cells

**Figure 7:**
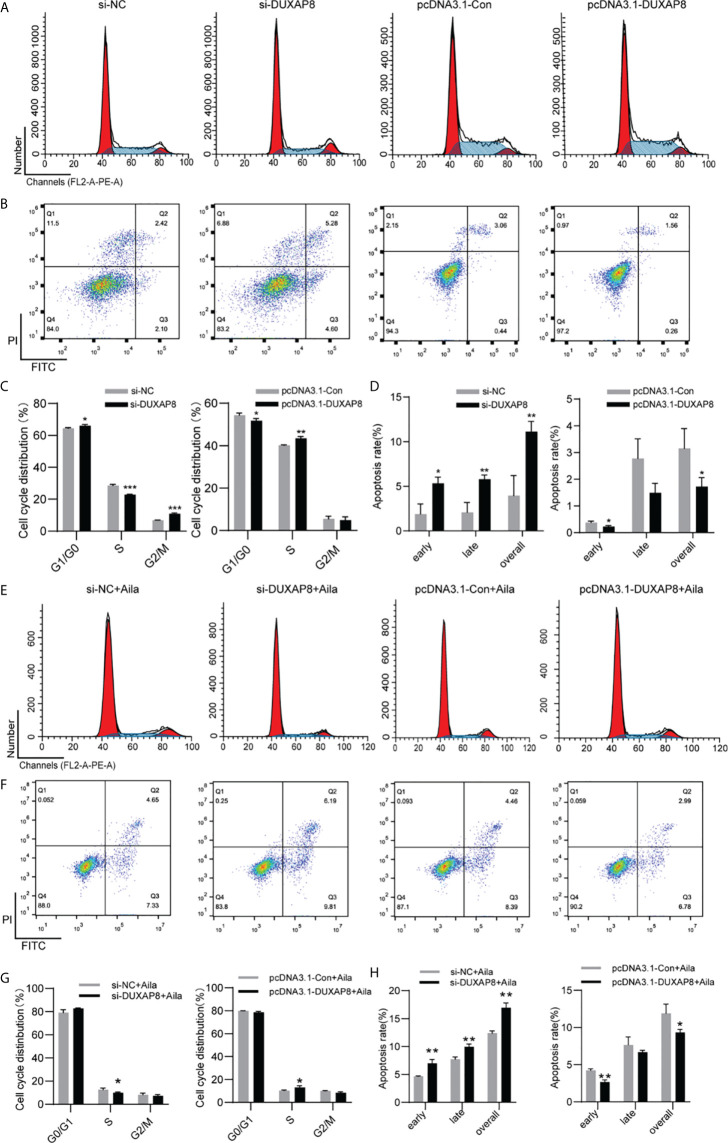
Analysis of the DUXAP8 expression on the cell cycle and apoptosis of H1299 cells. **(A)** The cell cycle was analyzed after si-DUXAP8 and pcDNA3.1-DUXAP8 transfection of H1299 cells. **(B)** Apoptosis was detected after si-DUXAP8 and pcDNA3.1-DUXAP8 transfection of H1299 cells. **(C)** Statistical analysis of the percentage of cell cycle distribution. **(D)** The quantified apoptosis data were calculated from the total proportion including both early and late apoptosis, the histogram represents the sum of Q2 and Q3. **(E)** The cell cycle was analyzed after si-DUXAP8 and pcDNA3.1-DUXAP8 transfection and treated with Aila. **(F)** Apoptosis was detected after si-DUXAP8 and pcDNA3.1-DUXAP8 and treated with Aila. **(G)** Statistical analysis of the percentage of cell cycle distribution. **(H)** The quantified apoptosis data were calculated from the total proportion including both early and late apoptosis, the histogram represents the sum of Q2 and Q3. The data are represented as the mean ± SD (n = 3). *(P < 0.05), **(P < 0.01) and ***(P < 0.001) indicate statistically significant differences.

To further analyze the effect of expression of *DUXAP8* on the cell cycle and apoptosis, H1299 cells was transfected with si-*DUXAP8* or pcDNA3.1-*DUXAP8* expression vectors and treated with Aila. Flow cytometry results showed that si-*DUXAP8* significantly decreased S phase, while pcDNA3.1-*DUXAP8* reversed this pattern in H1299 cells (**Figures 7A, C**). Cells treated with Aila and si-*DUXAP8* also significantly decreased S phase and overexpression of *DUXAP8* and Aila treated reversed this pattern (**Figures 7E, G**). To investigate whether *DUXAP8* is associated with apoptosis in H1299 cells, we used the Annexin V/PI double staining method. Results revealed that si-*DUXAP8* induced apoptosis of H1299 cells, while pcDNA3.1-*DUXAP8* reversed this effect (**Figures 7B, D**). Cells treated with Aila and si-*DUXAP8* also significantly induced apoptosis and overexpression of *DUXAP8* and Aila treated reversed this pattern (**Figures 7F, H**). These results were all consistent with our previous finding that *DUXAP8* was downregulated in NSCLC cells treated with Aila, indicating that downregulation of *DUXAP8* may represent a potential therapeutic strategy for the treatment of NSCLC.

### Expression Patterns of *DUXAP8* and *EGR1*

**Figure 8:**
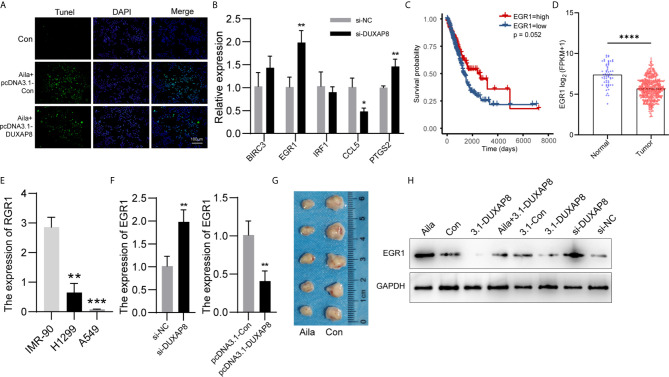
Expression patterns of *DUXAP8* and EGR1 in A549 cells. **(A)** The effect of *DUXAP8* overexpression on cell apoptosis after Aila treatment by tunnel tests in A549 cells. **(B)** Relative expression of PTGS2, IRF1, EGR1, BIRC3 and CCL5 after si-*DUXAP8* transfection by RT-PCR in A549 cells. **(C)** The effect of EGR1 expression level on patient survival in TCGA database. **(D)** Expression of EGR1 in LUAD based on sample types. **(E)** The expression of ERG1 between IMR-90, A549 and H1299 cells in CCLE database. **(F)** Relative mRNA expression of EGR1 in the Nc, si-*DUXAP8*, pcDNA3.1-Con, and pcDNA3.1-*DUXAP8* groups determined by RT-PCR in A549 cells. **(G)** Tumor morphology of the control group (right) and Aila group (left). **(H)** Relative protein expression of EGR1 in the Nc, si-*DUXAP8*, pcDNA3.1-Con, pcDNA3.1-*DUXAP8* and Aila treatment groups using western blotting in A549 cells. The data are represented as the mean ± SD (n = 3). *(P < 0.05), **(P < 0.01) ***(P < 0.001), and ****(P < 0.0001) indicate statistically significant differences.

We detected the effect of *DUXAP8* overexpression on cell apoptosis after Aila treatment *via* tunnel test. The results showed that overexpressing *DUXAP8* after Aila treatment can reduce the apoptosis (**Figure 8A**), indicating that *DUXAP8* may play an important role during the cell apoptosis. Based on the sequencing results, we screened *PTGS2*, *IRF1*, *EGR1*, *BIRC3* and *CCL5* genes, which are closely related to cell proliferation, cell cycle progression and apoptosis. First, RT-PCR was used to detect mRNA expression of these genes after knockdown of *DUXAP8*, and *EGR1* was significantly upregulated (**Figure 8B**). Moreover, TCGA database revealed that *EGR1* in lung adenocarcinoma patients (LUAD) was significantly lower than in noncancerous tissue, and the downregulation level of *EGR1* was associated with poor prognosis and short survival inversely (**Figures 8C, D**). CCLE database revealed that *EGR1* in A549 and H1299 cells was significantly lower than in IMR-90 cells (**Figure 8E**). Next, we transfected the knockdown and overexpression vectors to detect the interaction mechanism between *DUXAP8* and *EGR1* at the mRNA and protein levels. The results revealed that after knockdown of *DUXAP8*, expression of *EGR1* was much higher than in the control group by both RT-PCR and western blot analysis; in contrast, expression levels of *EGR1* were significantly decreased in the overexpression group (**Figures 8F, H**). Compared to the control group, Aila group delay the growth of tumor xenografts in mouse models (**Figure 8G**). This finding demonstrates that *EGR1* expression is regulated by *DUXAP8* and is negatively correlated with its expression.

## Discussion

In recent years, lncRNAs have become an attractive research focus because lncRNAs have been found to be involved in important physiological and pathological processes in a variety of cells. By interacting with multiple DNAs, RNAs and proteins, lncRNAs exhibit tumor-suppressive or oncogenic effects and have enormous potential as cancer biomarkers (46). It has already been demonstrated that abnormal expression of lncRNAs is closely related to the occurrence, metastasis, diagnosis and treatment of lung cancer (47). For example, EPEL promotes lung cancer cell proliferation by activating E2F (48). Additionally, MetaLnc9 facilitates metastasis of lung carcinoma by sensitizing cells to the AKT/mTOR signaling pathway (49). Furthermore, Li et al. found that AFAP1-AS1 was easily detected *in vivo*, which may help in diagnosing carcinoma (50). In addition, MALAT1 directly reverses the resistance of NSCLC cells to chemotherapeutic agents (51).

In our study, high-throughput lncRNA sequencing was conducted. The results showed that *DUXAP8* was significantly downregulated in H1299 cells treated with Aila. Moreover, knockdown of *DUXAP8* greatly inhibited cell viability and induced cell cycle arrest and apoptosis in A549 and H1299 cells, whereas overexpression of *DUXAP8* reversed these effects. Previous experiments reported that *DUXAP8* was overexpressed in cancers and that its aberrant upregulation promoted cancer cell growth (36), which is consistent with our results. Although the degree of apoptosis cycle arrest after overexpression and knockdown of *DUXAP8* was not completely consistent between A549 and H1299 cells, the Alia induced apoptosis and cell cycle arrest was substantially similar. Therefore, reduced expression of *DUXAP8* plays a vital role in restraining NSCLC.

An increasing number of reports suggest that decreased expression of *EGR1* is involved in cancer progression (52). Downregulation of *EGR1* contributes to the proliferation of colorectal cancer (42). SUN et al. performed RIP assays, and showed that *DUXAP8* RNA could directly bind to EZH2 in H1299 cells. Additionally, they found that EZH2 could directly bind to *EGR1* promoter region, and *DUXAP8* was able to repress *EGR1* by interacting with EZH2 (53). Our results demonstrated that *EGR1* is upregulated in response to knockdown of *DUXAP8*, inhibiting lung cancer growth.

Currently, approximately 75% of NSCLC patients in the world are at an advanced stage when diagnosed, leading to a particularly short life expectancy (8). As a malignant tumor, NSCLC has a complicated pathogenesis. Although a large number of studies have developed drugs to combat NSCLC, such as PD-1 inhibitors and angiogenesis inhibitors (54, 55), they are far from meeting the clinical demands. Therefore, it remains urgent to explore novel regulators to identify new therapeutic strategies for NSCLC. Natural products have been proven to possess powerful anticancer ability by regulating multiple genes and proteins related to cancers. Aila, an active compound extracted from Ailanthus altissima, has been shown to have a powerful inhibitory effect on NSCLC (13). However, there have been no studies investigating the relationships between Aila and *DUXAP8*.

Aila can significantly decrease cell viability of both B16 and A375, with the IC50 values of 1.83 and 5.77 μM (25). Aila is able to repress the viability of SGC-7901 cells and the IC50 at 72 h was 2.47 µM (56) and Ni et al. found that Aila has ability to inhibit A549 cell proliferation at 1.25uM (24). Based on these previous data, we decided to employ Aila with a concentration at 1 μM for this study. In the present research, it was shown that Aila significantly inhibits A549 and H1299 cell proliferation both *in vitro* and induced cell cycle arrest and apoptosis. Meanwhile, sequencing results demonstrated that Aila markedly downregulated *DUXAP8* and upregulated *EGR1* in H1299 cells. Consequently, we conclude that Aila suppresses cell viability and induces cycle arrest and apoptosis in A549 and H1299 cells by downregulating *DUXAP8* and upregulating *EGR1* expression.

Altogether, this research verifies the antitumor effects of Aila in NSCLC and further illuminates its mechanism involving *DUXAP8* and *EGR1*. These data all suggest that *DUXAP8* has great potential for the diagnosis, treatment, and prognosis of NSCLC, and our results provide a feasible theoretical basis for subsequent studies.

## Data Availability Statement

The datasets presented in this study can be found in online repositories. The names of the repository/repositories and accession number(s) can be found in the article/**Supplementary Material**.

## Author Contributions

Study concept and design: YZ, XD, and BW. Acquisition of data: LC, CW, DM, HW, and SC. Analysis and interpretation of data: LC, XW, YT, YL, and HW. Drafting of the manuscript: CW. Critical revision of the manuscript for important intellectual content: YZ, XD, BW, TW, and YS. Reagents and material support: YZ, XD, and BW. Administrative, technical, and supervision: YZ, XD, and BW. All authors contributed to the article and approved the submitted version.

## Funding

This research was funded by the Jilin Scientific and Technological Development Program (grant number: 20200404085YY) and the Science and technology project of traditional Chinese medicine in Jilin Province (grant number: 2021139) to XD.

## Supplementary Material

The Supplementary Material for this article can be found online at: https://www.frontiersin.org/articles/10.3389/fonc.2021.652567/full#supplementary-material

## Conflict of Interest

The authors declare that the research was conducted in the absence of any commercial or financial relationships that could be construed as a potential conflict of interest.

## References

[1] Bray F Ferlay J Soerjomataram I Siegel RL Torre LA Jemal A. Global Cancer Statistics 2018: GLOBOCAN Estimates of Incidence and Mortality Worldwide for 36 Cancers in 185 Countries. Ca-a Cancer J Clin (2018) 68:394–424. 10.3322/caac.21492 30207593

[2] Feng R-M Zong Y-N Cao S-M Xu R-H. Current Cancer Situation in China: Good or Bad News From the 2018 Global Cancer Statistics? Cancer Commun (2019) 39. 10.1186/s40880-019-0368-6 PMC6487510 31030667

[3] Travis WD Brambilla E Nicholson AG Yatabe Y Austin JHM Beasley MB. The 2015 World Health Organization Classification of Lung Tumors. J Thoracic Oncol (2015) 10:1243–60. 10.1097/JTO.0000000000000630 26291008

[4] Osmani L Askin F Gabrielson E Li QK. Current WHO Guidelines and the Critical Role of Immunohistochemical Markers in the Subclassification of Non-Small Cell Lung Carcinoma (NSCLC): Moving From Targeted Therapy to Immunotherapy. Semin Cancer Biol (2018) 52:103–9. 10.1016/j.semcancer.2017.11.019 PMC5970946 29183778

[5] Hirsch FR Scagliotti GV Mulshine JL Kwon R Curran WJ Wu YL. Lung Cancer: Current Therapies and New Targeted Treatments. Lancet (2017) 389:299–311. 10.1016/S0140-6736(16)30958-8 27574741

[6] Reck M Rabe KF. Precision Diagnosis and Treatment for Advanced Non-Small-Cell Lung Cancer. New Engl J Med (2017) 377:849–61. 10.1056/NEJMra1703413 28854088

[7] Swanton C Govindan R. Clinical Implications of Genomic Discoveries in Lung Cancer. N Engl J Med (2016) 374:1864–73. 10.1056/NEJMra1504688 27168435

[8] Folch E Costa DB Wright J VanderLaan PA. Lung Cancer Diagnosis and Staging in the Minimally Invasive Age With Increasing Demands for Tissue Analysis. Trans Lung Cancer Res (2015) 4:392–403. 10.3978/j.issn.2218-6751.2015.08.02 PMC4549479 26380180

[9] Huang Y Yuan K Tang M Yue J Bao L Wu S. Melatonin Inhibiting the Survival of Human Gastric Cancer Cells Under ER Stress Involving Autophagy and Ras-Raf-MAPK Signalling. J Cell Mol Med (2021) 25:1480–92. 10.1111/jcmm.16237 PMC7875909 33369155

[10] Haddadin S Perry MC. History of Small-Cell Lung Cancer. Clin Lung Cancer (2011) 12:87–93. 10.1016/j.cllc.2011.03.002 21550554

[11] Newman DJ Cragg GM. Natural Products as Sources of New Drugs Over the Last 25 Years. J Natural Products (2007) 70:461–77. 10.1021/np068054v 17309302

[12] Xiang Y Guo Z Zhu P Chen J Huang Y. Traditional Chinese Medicine as a Cancer Treatment: Modern Perspectives of Ancient But Advanced Science. Cancer Med (2019) 8:1958–75. 10.1002/cam4.2108 PMC6536969 30945475

[13] Mousavi F Shahali Y Pourpak Z Majd A Ghahremaninejad F. Year-to-Year Variation of the Elemental and Allergenic Contents of Ailanthus Altissima Pollen Grains: An Allergomic Study. Environ Monit Assess (2019) 191. 10.1007/s10661-019-7458-4 31079225

[14] Gao W Ge S Sun J. Ailanthone Exerts Anticancer Effect by Up-Regulating miR-148a Expression in MDA-MB-231 Breast Cancer Cells and Inhibiting Proliferation, Migration and Invasion. Biomedicine Pharmacotherapy (2019) 109:1062–9. 10.1016/j.biopha.2018.10.114 30551356

[15] Wang R Lu Y Li H Sun L Yang N Zhao M. Antitumor Activity of the Ailanthus Altissima Bark Phytochemical Ailanthone Against Breast Cancer MCF-7 Cells. Oncol Lett (2018) 15:6022–8. 10.3892/ol.2018.8039 PMC5840722 29552229

[16] He Y Peng S Wang J Chen H Cong X Chen A. Ailanthone Targets p23 to Overcome MDV3100 Resistance in Castration-Resistant Prostate Cancer. Nat Commun (2016) 7. 10.1038/ncomms13122 PMC5159881 27959342

[17] Daga M Pizzimenti S Dianzani C Cucci MA Cavalli R Grattarola M. Ailanthone Inhibits Cell Growth and Migration of Cisplatin Resistant Bladder Cancer Cells Through Down-Regulation of Nrf2, YAP, and c-Myc Expression. Phytomedicine (2019) 56:156–64. 10.1016/j.phymed.2018.10.034 30668336

[18] Cucci MA Grattarola M Dianzani C Damia G Ricci F Roetto A. Ailanthone Increases Oxidative Stress in CDDP-resistant Ovarian and Bladder Cancer Cells by Inhibiting of Nrf2 and YAP Expression Through a Post-Translational Mechanism. Free Radical Biol Med (2020) 150:125–35. 10.1016/j.freeradbiomed.2020.02.021 32101771

[19] Zhuo Z Hu J Yang X Chen M Lei X Deng L. Ailanthone Inhibits Huh7 Cancer Cell Growth *via* Cell Cycle Arrest and Apoptosis *In Vitro* and *In Vivo* . Sci Rep-Uk (2015) 5. 10.1038/srep16185 PMC4630794 26525771

[20] Chen Y Zhu L Yang X Wei C Chen C He Y. Ailanthone Induces G(2)/M Cell Cycle Arrest and Apoptosis of SGC-7901 Human Gastric Cancer Cells. Mol Med Rep (2017) 16:6821–7. 10.3892/mmr.2017.7491 PMC5865840 28901518

[21] Wei C Chen C Cheng Y Zhu L Wang Y Luo C. Ailanthone Induces Autophagic and Apoptotic Cell Death in Human Promyelocytic Leukemia HL-60 Cells. Oncol Lett (2018) 16:3569–76. 10.3892/ol.2018.9101 PMC6096173 30127963

[22] Zhang Y Zhang C Min D. Ailanthone Up-Regulates miR-449a to Restrain Acute Myeloid Leukemia Cells Growth, Migration and Invasion. Exp Mol Pathol (2019) 108:114–20. 10.1016/j.yexmp.2019.04.011 31002772

[23] Hou S Cheng Z Wang W Wang X Wu Y. Ailanthone Exerts an Antitumor Function on the Development of Human Lung Cancer by Upregulating Microrna-195. J Cell Biochem (2019) 120:10444–51. 10.1002/jcb.28329 30565729

[24] Ni Z Yao C Zhu X Gong C Xu Z Wang L. Ailanthone Inhibits Non-Small Cell Lung Cancer Cell Growth Through Repressing DNA Replication Via Downregulating RPA1. Br J Cancer (2017) 117:1621–30. 10.1038/bjc.2017.319 PMC5729430 29024939

[25] Liu W Liu X Pan Z Wang D Li M Chen X. Ailanthone Induces Cell Cycle Arrest and Apoptosis in Melanoma B16 and A375 Cells. Biomolecules (2019) 9. 10.3390/biom9070275 PMC6681521 31336757

[26] Yang P Sun D Jiang F. Ailanthone Promotes Human Vestibular Schwannoma Cell Apoptosis and Autophagy by Downregulation of Mir-21. Oncol Res (2018) 26:941–8. 10.3727/096504018X15149775533331 PMC7844645 29298734

[27] Kong D Ying B Zhang J Ying H. Retracted ArticleThe Anti-Osteosarcoma Property of Ailanthone Through Regulation of miR-126/VEGF-A Axis. Artif Cells Nanomedicine Biotechnol (2019) 47:3913–9. 10.1080/21691401.2019.1669622 31571500

[28] Kopp F Mendell JT. Functional Classification and Experimental Dissection of Long Noncoding Rnas. Cell (2018) 172:393–407. 10.1016/j.cell.2018.01.011 29373828 PMC5978744

[29] Rafiee A Riazi-Rad F Havaskary M Nuri F. Long Noncoding RNAs: Regulation, Function and Cancer. Biotechnol Genet Eng Rev (2018) 34:153–80. 10.1080/02648725.2018.1471566 30071765

[30] Chen ZY Lei TY Chen X Gu JY Huang JL Lu BB. Long Non-Coding RNA in Lung Cancer. Clin Chim Acta (2020) 504:190–200. 10.1016/j.cca.2019.11.031 31790697

[31] Li S Mei Z Hu HB Zhang X. The Lncrna MALAT1 Contributes to Non-Small Cell Lung Cancer Development Via Modulating miR-124/STAT3 Axis. J Cell Physiol (2018) 233:6679–88. 10.1002/jcp.26325 PMC13482422 29215698

[32] Tang Y Xiao GM Chen YJ Deng Y. Lncrna MALAT1 Promotes Migration and Invasion of Non-Small-Cell Lung Cancer by Targeting miR-206 and Activating Akt/mTOR Signaling. Anti-Cancer Drug (2018) 29:725–35. 10.1097/CAD.0000000000000650 29916897

[33] Zhao YJ Zhu ZX Shi SM Wang J Li N. Long Non-Coding RNA MEG3 Regulates Migration and Invasion of Lung Cancer Stem Cells *via* miR-650/SLC34A2 Axis. BioMed Pharmacother (2019) 120. 10.1016/j.biopha.2019.109457 31585300

[34] Xia H Qu XL Liu LY Qian DH Jing HY. Lncrna MEG3 Promotes the Sensitivity of Vincristine by Inhibiting Autophagy in Lung Cancer Chemotherapy. Eur Rev Med Pharmaco (2018) 22:1020–7. 10.26355/eurrev_201802_14384 29509250

[35] Ma H Xie M Sun M Chen T Jin R Ma T. The Pseudogene Derived Long Noncoding RNA DUXAP8 Promotes Gastric Cancer Cell Proliferation and Migration *via* Epigenetically Silencing PLEKHO1 Expression. Oncotarget (2017) 8:52211–24. 10.18632/oncotarget.11075 PMC5581023 28881724

[36] Hu Y Zhang X Zai H Jiang W Xiao L Zhu Q. Lncrna DUXAP8 Facilitates Multiple Malignant Phenotypes and Resistance to PARP Inhibitor in HCC* via* Upregulating Foxm1. Mol Ther Oncolytics (2020) 19:308–22. 10.1016/j.omto.2020.10.010 PMC7701012 33313387

[37] He W Yu Y Huang W Feng G Li J. The Pseudogene Duxap8 Promotes Colorectal Cancer Cell Proliferation, Invasion, and Migration by Inducing Epithelial-Mesenchymal Transition Through Interacting With EZH2 and H3k27me3. Oncotargets Ther (2020) 13:11059–70. 10.2147/OTT.S235643 PMC7605666 33149618

[38] Chen M Zheng Y Xie J Zhen E Zhou X. Integrative Profiling Analysis Identifies the Oncogenic Long Noncoding RNA DUXAP8 in Oral Cancer. Anti-Cancer Drug (2020) 31:792–8. 10.1097/CAD.0000000000000936 32304409

[39] Yin D Hua L Wang J Liu Y Li X. Long Non-Coding Rna DUXAP8 Facilitates Cell Viability, Migration, and Glycolysis in Non-Small-Cell Lung Cancer *via* Regulating HK2 and LDHA by Inhibition of Mir-409-3p. Oncotargets Ther (2020) 13:7111–23. 10.2147/OTT.S243542 PMC7383025 32801745

[40] Boone D Qi Y Li Z Hann S. Egr1 Mediates p53-independent c-Myc-induced Apoptosis *via* a Noncanonical ARF-dependent Transcriptional Mechanism. Proc Natl Acad Sci USA (2011) 108:632–7. 10.1073/pnas.1008848108 PMC3021028 21187408

[41] Zhang H Chen X Wang J Guang W Han W Zhang H. EGR1 Decreases the Malignancy of Human Non-Small Cell Lung Carcinoma by Regulating KRT18 Expression. Sci Rep (2014) 4:5416. 10.1038/srep05416 24990820 PMC4080516

[42] Wei F Jing H Wei M Liu L Wu J Wang M. Ring Finger Protein 2 Promotes Colorectal Cancer Progression by Suppressing Early Growth Response 1. Aging (2020) 12:26199–220. 10.18632/aging.202396 PMC7803491 33346749

[43] Shi S Li F Wu L Zhang L Liu L. Feasibility of BMSCs Mediated-Synthetic Radiosensitive Promoter Combined NIS for Radiogenetic Ovarian Cancer Therapy. Hum Gene Ther (2020). 10.1089/hum.2020.214 33339472

[44] Yu G Wang LG Han Y He QY. clusterProfiler: An R Package for Comparing Biological Themes Among Gene Clusters. Omics (2012) 16:284–7. 10.1089/omi.2011.0118 PMC3339379 22455463

[45] Uhl E Wolff F Mangal S Dube H Zanin E. Light-Controlled Cell-Cycle Arrest and Apoptosis. Angewandte Chemie (International Ed English) (2020) 60:1187–96. 10.1002/anie.202008267 PMC7839536 33035402

[46] Iyer M Niknafs Y Malik R Singhal U Sahu A Hosono Y. The Landscape of Long Noncoding RNAs in the Human Transcriptome. Nat Genet (2015) 47:199–208. 10.1038/ng.3192 25599403 PMC4417758

[47] Qiu MT Feng DJ Zhang HT Xia WJ Xu YT Wang J. Comprehensive Analysis of lncRNA Expression Profiles and Identification of Functional lncRNAs in Lung Adenocarcinoma. Oncotarget (2016) 7:16012–22. 10.18632/oncotarget.7559 PMC4941294 26918601

[48] Park SM Choi EY Bae DH Sohn HA Kim SY Kim YJ. The LncRNA Epel Promotes Lung Cancer Cell Proliferation Through E2F Target Activation. Cell Physiol Biochem (2018) 45:1270–83. 10.1159/000487460 29448242

[49] Yu T Zhao YJ Hu ZX Li J Chu DD Zhang JW. Metalnc9 Facilitates Lung Cancer Metastasis *via* a PGK1-Activated Akt/mTOR Pathway. Cancer Res (2017) 77:5782–94. 10.1158/0008-5472.CAN-17-0671 28923857

[50] Wei C-C Nie F-Q Jiang L-L Chen Q-N Chen Z-Y Chen X. The Pseudogene DUXAP10 Promotes an Aggressive Phenotype Through Binding With LSD1 and Repressing LATS2 and RRAD in Non Small Cell Lung Cancer. Oncotarget (2017) 8:5233–46. 10.18632/oncotarget.14125 PMC5354904 28029651

[51] Jiang Y Sun A Zhao Y Ying W Sun H Yang X. Proteomics Identifies New Therapeutic Targets of Early-Stage Hepatocellular Carcinoma. Nature (2019) 567:257–61. 10.1038/s41586-019-0987-8 30814741

[52] Ma Z Gao X Shuai Y Wu X Yan Y Xing X. EGR1-Mediated linc01503 Promotes Cell Cycle Progression and Tumorigenesis in Gastric Cancer. Cell proliferation (2020) 54:e12922. 10.1111/cpr.12922 33145887 PMC7791171

[53] Sun M Nie FQ Zang C Wang Y Hou J Wei C. The Pseudogene Duxap8 Promotes Non-Small-Cell Lung Cancer Cell Proliferation and Invasion by Epigenetically Silencing EGR1 and RHOB. Mol Ther (2017) 25:739–51. 10.1016/j.ymthe.2016.12.018 PMC5363203 28131418

[54] Malapelle U Rossi A. Emerging Angiogenesis Inhibitors for Non-Small Cell Lung Cancer. Expert Opin Emerg Dr (2019) 24:71–81. 10.1080/14728214.2019.1619696 31092048

[55] Liu TT Ding SL Dang J Wang H Chen J Li G. First-Line Immune Checkpoint Inhibitors for Advanced Non-Small Cell Lung Cancer With Wild-Type Epidermal Growth Factor Receptor (EGFR) or Anaplastic Lymphoma Kinase (ALK): A Systematic Review and Network Meta-Analysis. J Thorac Dis (2019) 11:2899–+. 10.21037/jtd.2019.07.45 PMC6687998 31463119

[56] Chen Y Zhu L Yang X Wei C Chen C He Y. Ailanthone Induces G2/M Cell Cycle Arrest and Apoptosis of SGC−7901 Human Gastric Cancer Cells. Mol Med Rep (2017) 16:6821–7. 10.3892/mmr.2017.7491 PMC5865840 28901518

