# Supplementary material for: Analysis of Long Noncoding RNAs in Aila-Induced Non-Small Cell Lung Cancer Inhibition
Source: Front Oncol. 2021 Jun 21;11:652567. doi: 10.3389/fonc.2021.652567 (PMC8255921; doi:10.3389/fonc.2021.652567)
Supplement: Supplementary file 1 [file DataSheet_1.pdf]

Supplementary Information to

Analysis of long noncoding RNAs in Aila-induced non-small cell lung cancer inhibition

Lin Chen, Cui Wu, Heming Wang, Sinuo Chen, Danhui Ma, Ye Tao, Xingye Wang, Yanhe Luan, Tiedong Wang, Yan Shi, Guangqi Song, Yicheng Zhao, Xijun Dong, Bingmei Wang

To whom correspondence should be addressed:
yichengzhao@live.cn, dongxijun1064@163.com, bingmeiwang1970@163.com

Table of contents

Supplementary Figure. 1 | **Ailanthone Purity Report**

Supplementary Table. 1 | **Sequence of siRNAs**

Supplementary Table. 2 | **Sequence of Primers**

Supplementary Figure. 1 | **Ailanthone Purity Report**

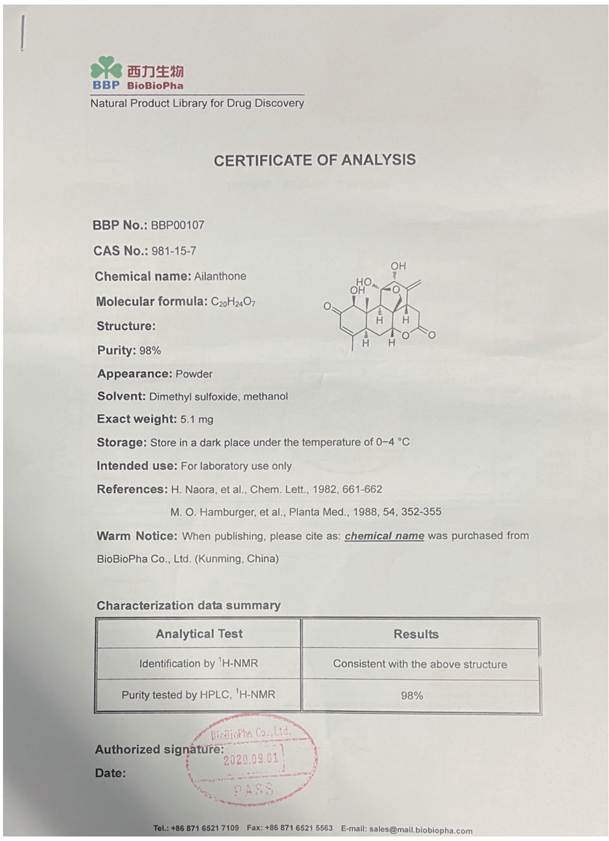

西力生物
BBP BioBioPha
Natural Product Library for Drug Discovery

CERTIFICATE OF ANALYSIS

BBP No.: BBP00107

CAS No.: 981-15-7

Chemical name: Ailanthone

Molecular formula: $C_{20}H_{24}O_7$

Structure:

Purity: 98%

Appearance: Powder

Solvent: Dimethyl sulfoxide, methanol

Exact weight: 5.1 mg

Storage: Store in a dark place under the temperature of 0–4 °C

Intended use: For laboratory use only

References: H. Naora, et al., Chem. Lett., 1982, 661-662
M. O. Hamburger, et al., Planta Med., 1988, 54, 352-355

Warm Notice: When publishing, please cite as: ***chemical name*** was purchased from BioBioPha Co., Ltd. (Kunming, China)

Characterization data summary

Analytical Test	Results
Identification by ^{1}H-NMR	Consistent with the above structure
Purity tested by HPLC, ^{1}H-NMR	98%

Authorized signature:

Date:

BioBioPha Co., Ltd. 2020.09.01 PASS

Tel.: +86 871 6521 7109 Fax: +86 871 6521 5563 E-mail: sales@mail.biobiopha.com

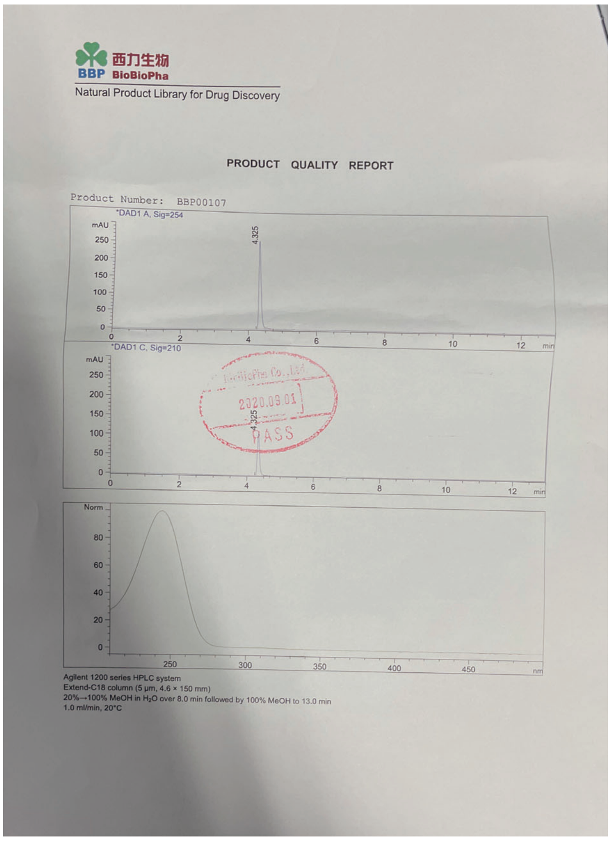

西力生物
BBP BioBioPha
Natural Product Library for Drug Discovery

PRODUCT QUALITY REPORT

Product Number: BBP00107

Agilent 1200 series HPLC system
Extend-C18 column (5 μm, 4.6 × 150 mm)
20%→100% MeOH in H_2O over 8.0 min followed by 100% MeOH to 13.0 min
1.0 ml/min, 20°C

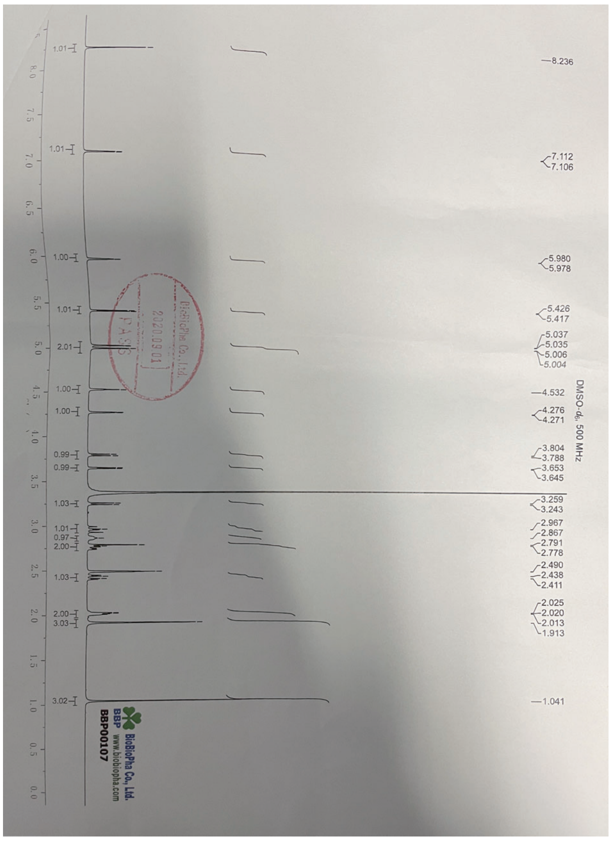

^{1}H-NMR and HPLC show the Ailanthone composite configuration and the purity (>98%).

Supplementary Table. 1 | **Sequence of siRNAs**

siRNA	Sequences
si-DUXAP8	AAGATAAAGGTGGTTTCCACAAGA

Supplementary Table. 2 | **Sequence of Primers**

Genes	Sequences
GAPDH-F	TGGTATCGTGGAAGGACTCA
GAPDH-R	GGGCCATCGACAGTCTTC
DUXAP8-F	AGGATGGAGTCTCGCTGTATTGC
DUXAP8-R	GGAGGTTTGTTTTCTTCTTTTTT
GARS1-DT-F	CCAGCCCCACAAGACTAAGG
GARS1-DT-R	GGAAACAATGGTCCCAGCAC
AL162595.1-F	GCTGGCACCACCTATAAGCA
AL162595.1-R	GCCTCAGTATCAGCAGTCCC
EGR1-F	GGTCAGTGGCCTAGTGAGC
EGR1-R	GTGCCGCTGAGTAAATGGGA
PTGS2-F	TGACCAGAGCAGGCAGATGA
PTGS2-R	CCAGTAGGCAGGAGAACATATAACA
IRF1- F	CGGGATCCATGCCCATCACTCGGATGCGCA
IRF1- R	CCGGAATTCCTACGGTGCACAGGGAA
BIRC3-F	CCAAGTGGTTTCCAAGGTGT
BIRC3-R	TGGGCTGTCTGATGTGGATA
CCL5-F	AATCTTGCAGTCGTGTTTGTCA
CCL5-R	AGCTCATCTCCAAATAGTTG ATGT
